# *In Vitro* Degradation of Plasticized PLA Electrospun Fiber Mats: Morphological, Thermal and Crystalline Evolution

**DOI:** 10.3390/polym12122975

**Published:** 2020-12-13

**Authors:** Adrián Leonés, Laura Peponi, Marcela Lieblich, Rosario Benavente, Stefano Fiori

**Affiliations:** 1 Instituto de Ciencia y Tecnología de Polímeros (ICTP-CSIC), C/Juan de la Cierva 3, 28006 Madrid, Spain; aleones@ictp.csic.es (A.L.); rbenavente@ictp.csic.es (R.B.); 2 Interdisciplinary Platform for “Sustainable Plastics towards a Circular Economy” (SUSPLAST-CSIC), 28006 Madrid, Spain; 3 Centro Nacional de Investigaciones Metalúrgicas (CENIM-CSIC), 28040 Madrid, Spain; marcela@cenim.csic.es; 4 Condensia Química SA, R&D Department, C/La Cierva 8, 08184 Barcelona, Spain; s.fiori@condensia.com

**Keywords:** PLA, electrospun fibers, hydrolytic degradation, plasticizer, crystallinity evolution

## Abstract

In the present work, fiber mats of poly(lactic acid), PLA, plasticized by different amounts of oligomer lactic acid, OLA, were obtained by electrospinning in order to investigate their long term hydrolytic degradation. This was performed in a simulated body fluid for up to 352 days, until the complete degradation of the samples is reached. The evolution of the plasticized electrospun mats was followed in terms of morphological, thermal, chemical and crystalline changes. Mass variation and water uptake of PLA-based electrospun mats, together with pH stability of the immersion media, were also studied during the *in vitro* test. The results showed that the addition of OLA increases the hydrolytic degradation rate of PLA electrospun fiber mats. Moreover, by adding different amounts of OLA, the time of degradation of the electrospun fiber mats can be modulated over the course of a year. Effectively, by increasing the amount of OLA, the diameter of the electrospun fibers decreases more rapidly during degradation. On the other hand, the degree of crystallinity and the dimension of the α crystals of the electrospun fiber mats are highly affected not only by the presence but also by the amount of OLA during the whole process.

## 1. Introduction

The electrospinning process is a versatile technique which offers a wide range of options that can be modified in order to obtain electrospun polymeric fibers with different final properties [1]. Some strategies have been studied in order to tailor the thermal, mechanical and crystalline properties of electrospun polymeric fibers such as blending with other polymers or plasticizers to modulate their glass transition temperature (*T*_g_) [2], reinforcing with nanoparticles for improving their mechanical performance [3,4] or electrospinning post-processing to modify their final properties [5]. Electrospinning makes it possible to obtain randomly or oriented fibers at the micron/nano-scale, with large surface areas and high porosity, with applications in the food packaging [6,7,8], tissue engineering [9], smart textile [10], energy [11], and air filtration [12], or high–performance engineering fields [8,13]. Thanks to its versatility, its low cost and its potential scalability to the industrial level [14], electrospinning has been consolidated as a suitable way to obtain advanced polymeric fibers.

Specifically, for the biomedical application field, biopolymers have to be used. Among them, poly(lactic acid), PLA, is a non-petrochemical based polymer, widely studied due to its biocompatibility [15] and degradability into non-toxic products in physiological conditions [16].

However, PLA is very brittle, showing poor mechanical performance in terms of tensile strength and elongation at break [17]. For this reason, many studies are focused on the improvement of the flexibility of PLA by using plasticizer [18] or copolymerizing it with other more elastic polymers [19]. Nevertheless, when using plasticizer, a good miscibility between the polymer and plasticizer has to be achieved. Previous works have been conducted on the use of oligomer lactic acid (OLA) as a plasticizer in PLA. Both materials belong to the same family of aliphatic polyesters, contributing to their good compatibility [20,21].

Many studies have been carried out on the hydrolytic degradation of PLA in bulk, focusing on factors that can influence the degradation rate such as degree of crystallinity [22], molecular weight [23], temperature and pH of surrounding media [16], etc. All these factors strongly affect the time that PLA needs to totally degrade in physiological conditions, which is one of the main properties to take into account for potential applications such as biomedical implants. In terms of the difference of bulk PLA materials, very few scientific articles reported studies on the hydrolytic degradation of PLA electrospun fiber mats. In addition, in these cases, very short degradation times are taken into account without achieving the total degradation of the electrospun fiber mats. Rowe et al. [15] reported the degradation of PLA-electrospun fibers reinforced with bioglass, in phosphate buffered saline (PBS) during 9 days of immersion. Nim et al. [24] studied the photo-degradation of PLA electrospun fibers reinforced with TiO_2_ samples in PBS during a period of 7 days, reporting changes in the morphology of the fibers after 4 days of immersion. In particular, considering the potential application of PLA electrospun fibers as biodegradable implants [1], our present work is focused on the study of degradation in simulated physiological condition at 37 °C, during a period of almost one year, until reaching their complete degradation. Based on our knowledge, this is the first time that an *in vitro* degradation test has been reported until the complete disintegration of the PLA-based electrospun fiber mats has been reached. Moreover, the properties of PLA electrospun fiber mats are tuned by the addition of OLA as plasticizer at different concentrations, correlating their degradation process with the different amounts of OLA. In particular, we focus on the changes in the morphology of the electrospun fibers observed by scanning electron microscopy (SEM) and their thermal properties measured by differential scanning calorimetry (DSC), pointing out the chemical changes during the hydrolytic degradation process as well as the crystallinity evolution, studied by X-ray diffraction (XRD) and attenuated total reflectance-Fourier transform infrared spectroscopy (ATR-FTIR). Finally, hydrolytic degradation process was studied in terms of pH changes, mass variation and water uptake of PLA-based electrospun mats during the *in vitro* test.

## 2. Materials and Methods

### 2.1. Materials

Polylactic acid (PLA3051D, 3% of D-lactic acid monomer, molecular weight 142 × 10^4^ g·mol^−1^, density 1.24 g·cm^−3^) was supplied by NatureWorks^®^. Lactic acid oligomer (Glyplast OLA8, ester content >99%, density 1.11 g·cm^−3^, viscosity 22.5 mPa.s, molecular weight 1100 g·mol^−1^) was kindly supplied by Condensia Quimica SA. PLA:OLA electrospun fiber mats were prepared in an Electrospinner Y-flow 2.2.D-XXX (Nanotechnology Solutions) following our previously reported protocol [25,26,27], working conditions of 22% of humidity and a temperature of 20 °C. Briefly, the polymers are solved in a solvent solution of chloroform:dimethilformamide, in a proportion of 4:1, overnight at ambient conditions. Then, the optimization of the main parameters of the electrospinning process were achieved and a voltage of 20 V, a distance of 17 cm between tip and collector and a polymer solution flux of 3.5 mL/h were used in order to obtain randomly-oriented electrospun fiber mats. Different concentrations of OLA have been used, in order to study how the OLA amount can affect the final properties of the electrospun mats during the degradation process in terms of morphology, thermal properties and crystallinity. In particular, 10, 20 and 30 wt% of OLA was added to PLA, named PLA90, PLA80 and PLA70, respectively. PLA indicates electrospun fiber mats of 100% PLA, as indicated in Table 1.

### 2.2. Characterization Techniques

Scanning Electron Microscopy, SEM, (PHILIPS XL30 Scanning Electron Microscope) was used in order to study the morphology and diameter evolution of the electrospun fibers. All the samples were previously gold-coated (~5 nm thickness) in a Polaron SC7640 Auto/Manual Sputter. SEM image analysis was carried out with ImageJ software. Diameters were calculated as the average value of 30 random measurements for each sample. Energy-dispersive X-ray spectroscopy (EDX) analyses were carried out using Hitachi SU 8000 FE-SEM equipment with a Bruker XFlash Detector 5030 working at 15 Kv.

The dynamic viscosity of the electrospun polymeric solutions was determined using an AR-G2 TA Instruments rheometer with parallel plate geometry (40 mm in diameter). Rotational tests were conducted using a stepped shear rate from 0.01 to 1500 s^−1^ at 20 °C.

The *in vitro* degradation process was studied by immersing squares of 1 cm^2^ of each PLA: OLA electrospun fiber mat, supported by their aluminum foil, in Phosphate Buffered Saline solution (PBS) for 352 days, taking samples at 1, 3 and 7 days to characterize their evolution during the first week, taking samples at 14, 21 and 28 days, to characterize the first month, at 84 days, corresponding to 3 months, up to 352 days, representing almost 1 year of the experiment. The extraction days are named *T*_x_, where x indicates the number of the corresponding day. The as-obtained electrospun mats are considered as time 0, T0, and are used as references. Each week, the PBS solution was renovated. The *in vitro* degradation process was run in an oven at a constant temperature of 37 ± 1 °C.

The pH evolution was measured every 7 days for 352 days with a pH METER-02 (Homtiky) with an error of ± 0.01.

The mass of the PLA-based electrospun fiber samples was measured before beginning the immersion in PBS and after each extraction day. Samples were removed from the solution, their surface was dried with a paper towel and was weighted obtaining the “wet samples weight”. Then, samples were dried for 2 weeks under vacuum; afterwards, their weight was measured, obtaining the “dry samples weight”. Water accumulation was obtained from the difference between the mass of wet and dried samples. The mass loss was calculated from the difference between the mass of the dried samples and the mass of the initial samples. A precision balance was used to weight all samples within an error of 0.05 mg. The results are given in mass % with respect to the initial mass for each sample.

(1) $$\mathrm{Water}\;\mathrm{uptake}\;\%=\;100\times \left(\frac{\mathrm{Weight}\;\mathrm{wet}-\mathrm{Weight}\;\mathrm{dry}}{\mathrm{Weight}\;\mathrm{dry}}\right)$$

(2) $$\mathrm{Mass}\;\mathrm{variation}\;\%=\;100\times \left(\frac{\mathrm{Weight}\;\mathrm{dry}-\mathrm{Weight}\;\mathrm{initial}}{\mathrm{Weight}\;\mathrm{initial}}\right)$$

Attenuated total reflectance-Fourier transform infrared spectroscopy (ATR-FTIR) measurements were conducted by a Spectrum One FTIR spectrometer (Perkin Elmer instruments). Spectra were obtained in the 4000–400 cm^−1^ region at room temperature in transmission mode with a resolution of 4 cm^−1^.

Thermal transitions were studied by Differential Scanning Calorimetry in a DSC Q2000 TA instrument under nitrogen atmosphere (50 mL·min^−1^). The thermal analysis was programmed at 10 °C.min^−1^ from −60 °C up to 180 °C, obtaining the glass transition temperature (*T*_g_) that was calculated as the midpoint of the transition, the cold crystallization enthalpy (Δ*H*_cc_) and the melting enthalpy (Δ*H*_m_). The degree of crystallinity (*X*_c_%) was calculated using Equation (3), taking the value of crystallization enthalpy of pure crystalline PLA (Δ*H*_m_^0^) as 93.6 J·g^−1^ and *W*_f_ as the weight fraction of PLA in the sample [28].

(3) $$X_{c}\%=\;100\times \left(\frac{\Delta H_{m}-\Delta H_{\mathrm{cc}}}{\Delta H_{m}^{0}}\right)\times \frac{1}{W_{f}}$$

Wide-Angle X-ray Diffraction (WAXD) measurements were performed using a Bruker D8 Advance instrument with a CuK as source (0.154 nm) and a Detector Vantec1. The scanning range was 5° and 60°, and the step-size and count time per step were 0.023851° and 0.5 s, respectively.

Furthermore, from the WAXD data, the crystal size in the (*hkl*) direction can be obtained through the Scherrer equation 4:

(4) $$D=\;\frac{k\lambda }{\beta \cos\left(\theta \right)}$$

In the Equation (4), D is the crystal size, *k* is the shape factor with a typical value of 0.9 [19,29], *λ* is the wavelength of the incident wave, *β* is the broadening of the peak at half of the maximum peak and *θ* is the diffraction angle.

## 3. Results and Discussion

**Figure 1:**
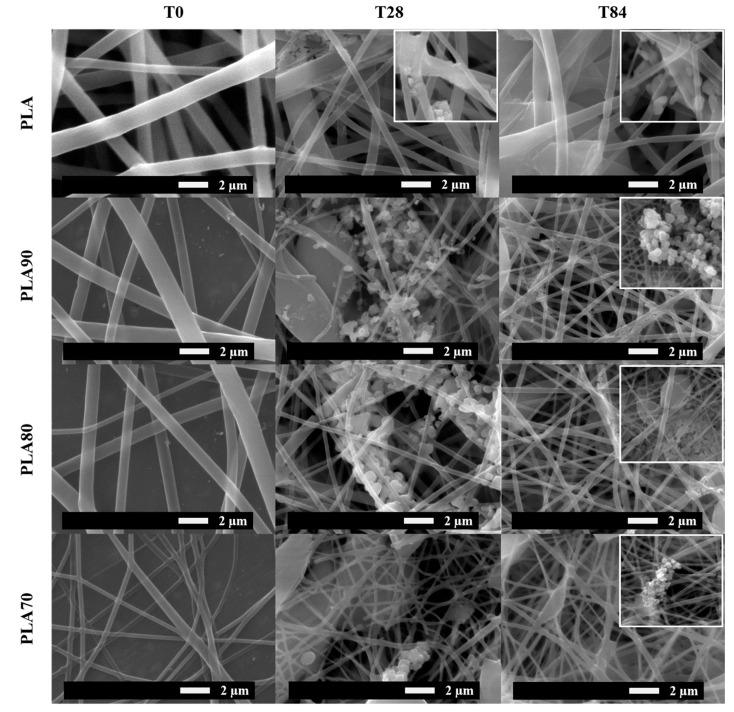
SEM images at 8000× of neat PLA and each PLA: OLA electrospun samples after 0, 28 and 84 days of immersion in PBS.

Once randomly oriented electrospun PLA-based fiber mats are obtained, the morphologies of neat PLA and PLA:OLA electrospun fiber mats are studied at different times of degradation in PBS by SEM analysis, starting from the as-obtained mats, which are equivalent to time 0 of degradation (T0). In Figure 1, SEM images for neat PLA and PLA:OLA 70:30, 80:20 and 90:10 (named PLA70, PLA80 and PLA90, respectively) are reported at two times of degradation, 28 (T28) and 84 (T84) days, and compared with the as-obtained mats (T0). For all the samples, straight, randomly oriented fibers with homogenous bead-free morphology can be observed before degradation starts. In particular, at T0, the surface of nanofibers looks smooth with average diameters of 904 ± 33 nm for neat PLA, 875 ± 63 nm for PLA90, 689 ± 34 nm for PLA80 and 476 ± 80 nm for PLA70, as summarized in Table 1.

In agreement with the diameter evolution, the same evolution in the solution viscosity of the different samples is observed. In fact, referring to the viscosity of PLA solution, the addition of 10 wt% of OLA decreases the viscosity of the solution by 5%, up to a decrease of 55% with respect to the viscosity of PLA when 30 wt% of OLA is added. These values are in good agreement with those reported in Arrieta et al. [30] for blends based on PLA and PHB plasticized with OLA.

After 1 month (T28) of immersion in PBS media, a mineralization process during degradation can be observed in all the PLA-based electrospun fiber mats. The minerals formed from solution showed similar spherical morphology in all the PLA-based samples. Moreover, in all the cases, after 28 days of immersion in PBS, the electrospun fibers show a reduction in the average diameter dimension of more than 50%.

After 3 months of degradation (T84), a considerable number of holes on the surface of nanofibers can be observed in all the samples. At this time, the average diameter of fibers decreased to 433 ± 114 nm for neat PLA, 303 ± 81 nm for PLA90, 215 ± 53 nm for PLA80 and 175 ± 45 nm for PLA70, respectively. These values correspond to a reduction in the average diameter of 52% for neat PLA, 65% for PLA90, 69% for PLA80 and 64% for PLA70, indicating that by increasing the amount of OLA, the diameter reduction increases during the degradation process, being quite constant for PLA and PLA90 with respect to the first month of degradation.

**Figure 2:**
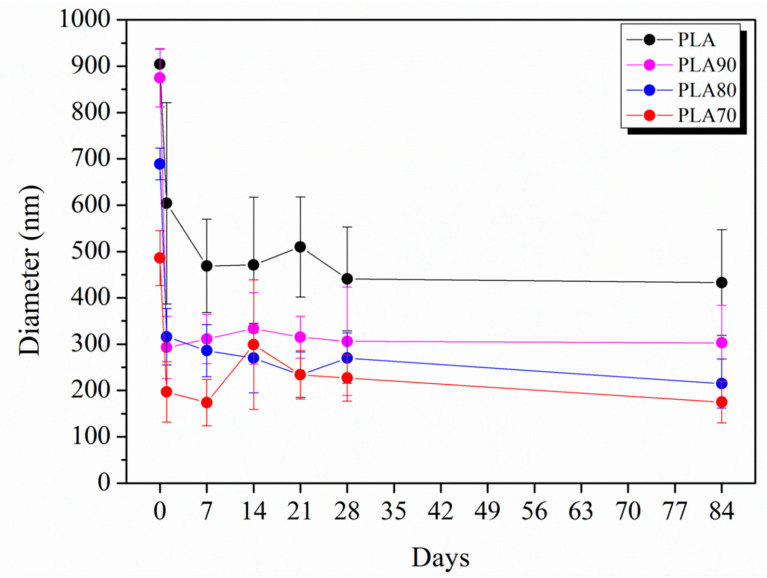
Diameter evolution measured by SEM of neat PLA and each PLA:OLA electrospun samples by increasing the days of degradation in PBS.

The evolution of the average diameter of PLA:OLA electrospun fiber mats by increasing the immersion time in PBS is shown in Figure 2, where the average diameters are reported for 1, 7, 14, 21, 28 and 84 immersion days, named T1, T7, T14, T21, T28 and T84, respectively. For PLA electrospun fiber mats, after 1 day of immersion, the average diameter slightly decreased from 904 ± 33 nm to 604 ± 217 nm, and the highest decrease in diameter was observed after 7 days with a value of 469 ± 101 nm, a 48% reduction, which remained approximately constant until 84 days of immersion in PBS. Similar behavior was observed for all the PLA:OLA systems with a slight decrease after 1 day of immersion and a pronounced reduction after the first week, which remained constant during all the duration of the degradation test. In particular, after the first 7 days of immersion in PBS, a reduction in diameter of 63% for PLA90, 58% for PLA80 and 64% for PLA70 was observed.

Furthermore, the mineralization process observed by SEM was corroborated by EDX analysis (see Table 2). In fact, looking at the element composition obtained by EDX, we can be certain that the degradation process in PBS media leads to the presence of Cl and Na on the electrospun mats. Moreover, by X-Ray analysis, three clear peaks can be identified in all the PLA-based samples at 2θ = 32, 45.5 and 56.5°, which are attributed to the [200], [220] and [222] crystallographic planes of NaCl [31].

**Figure 3:**
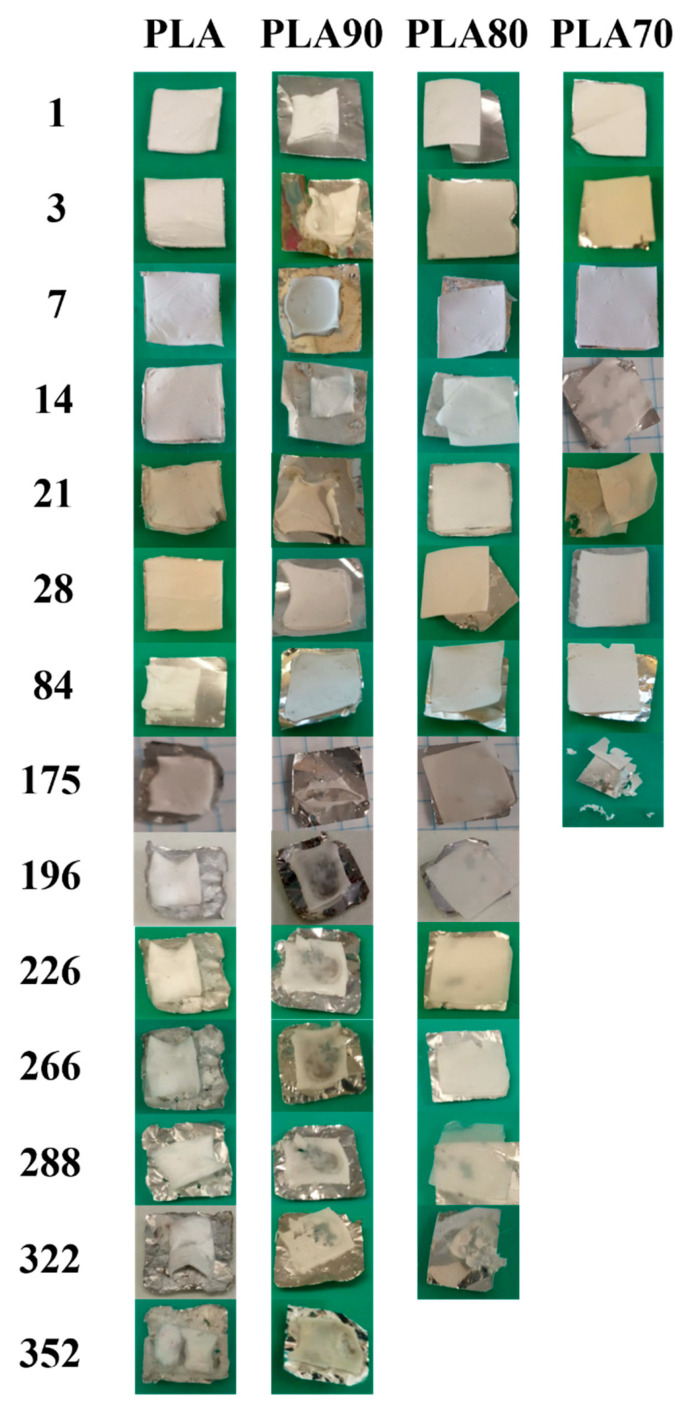
Visual appearance of neat PLA and each of the PLA:OLA electrospun samples after different amounts of days of immersion in PBS.

We decided to select T84 as the last day of degradation for studying and comparing the properties of the electrospun mats. At this time, all the samples still maintained their fiber structure, as can be seen in Figure 3, where the visual appearance of the different mats during the entire degradation process is shown. In fact, after more than 84 days of immersion in PBS, the samples started to break, and in the case of PLA70 it is worth noting its disintegrability at 175 days.

Furthermore, from the visual appearance of the degraded mats, their reduction in size is observed at different times, depending on their composition. PLA90 shows a significant reduction in size from the first day of degradation. This behavior is found to remain constant throughout the degradation process for this sample. For the other samples, the reduction in size of the mat is observed later, with PLA80 decreasing its size starting from day 7, and PLA70 showing a reduction in size after 21 days of degradation in PBS. Finally, for neat PLA, the electrospun mat retained its morphology until day 84, when a reduction was observed, with this being the slowest sample to show this behavior. On the other hand, by increasing the time of degradation, all electrospun mats show clear signs of color change and increases in transparency. This is particularly evident in PLA80 and PLA90, where from day 196 and 288, respectively, it was possible to see the aluminum foil through the samples due to its high transparency; this is attributed to a delamination process. As said before, the first sample to achieve the total degradation was PLA70. PLA80 needed 322 days to reach its disintegration. At the same day, PLA90 was broken into pieces of different dimensions while PLA maintained its integrity even with high transparency and delamination. These results show that by increasing the amount of OLA, the disintegration in PBS of the electrospun mats occurs faster. Moreover, these results are very interesting considering that, normally, when a degradation study is carried out, only the first months are considered, and it is quite difficult to find experiments carried out until the total degradation of the samples is reached, which in our case occurred after one year of immersion in PBS. Furthermore, it is well known that the use of plasticizer can affect the final properties of the materials, especially in terms of migration and of color changes [32,33]. By using OLA plasticizer, we have not found any color change in our electrospun samples.

**Figure 4:**
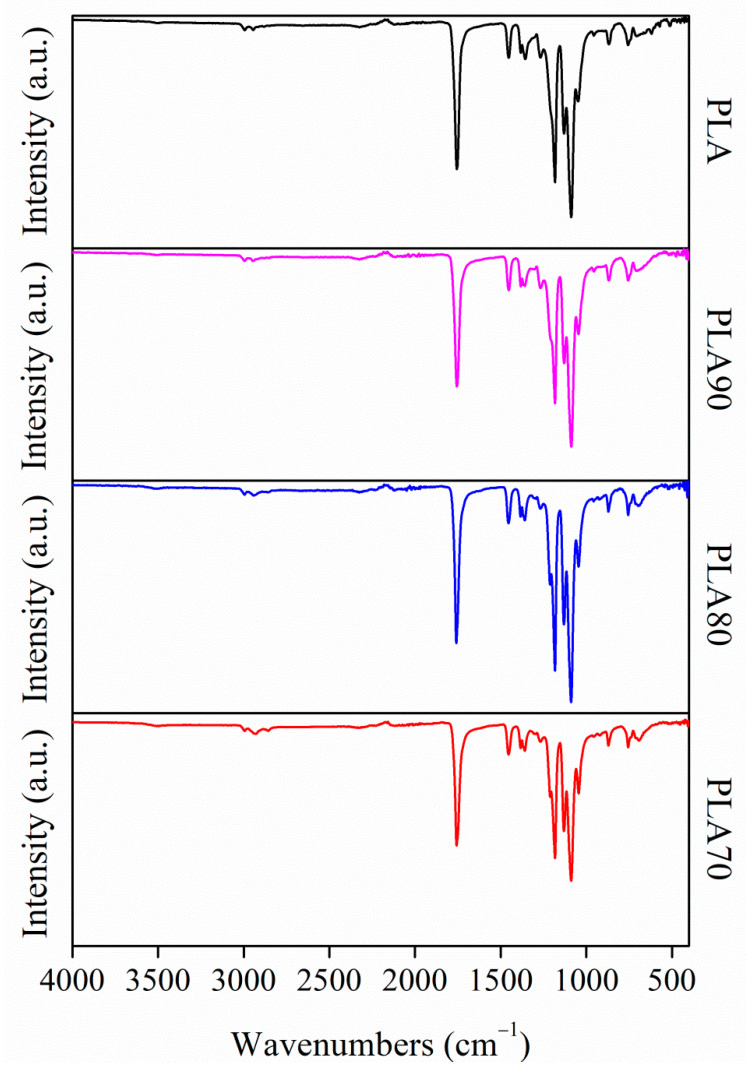
FTIR spectra in the 4000–400 cm^−1^ range for PLA and PLA:OLA electrospun samples.

In order to deeply study the degradation process, the chemical structure of the samples must be considered at T0 and during the different degradation steps. Figure 4 shows the FT-IR spectra, in the 4000–400 cm^−1^ range, of all the PLA:OLA electrospun samples at T0.

The spectra display the typical bands of PLA for each of the electrospun mats. The stretching of the carbonyl group (–C=O) at 1750 cm^−1^, which it is attributed to the amorphous carbonyl vibration, is observed. The CH_3_ asymmetric deformation mode around 1453 cm^−1^ and the CH_3_ symmetric deformation mode around 1386 cm^−1^ can be observed in all the samples. Furthermore, two frequency bands (2995 and 2940 cm^−1^) can be observed, which are attributed to ν_as_ (CH_3_) and ν_s_ (CH_3_), respectively. Finally, at 1080 cm^−1^, a band associated with the –C–O– asymmetric mode is presented, as well as the band at 1360 cm^−1^, which is related to the CH deformation.

**Figure 5:**
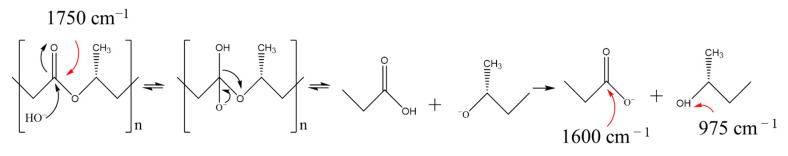
Hydrolytic chain cleavage mechanism of PLA [34] and the most significant infrared bands for monitoring the reaction by FTIR.

PLA belongs to the family of aliphatic polyesters, as mentioned previously, which means that its ester groups are susceptible to being hydrolytically degraded in the physiological environment according to reaction shown in Figure 5. The hydrolysis of the ester groups leads to the breaking of the polymer chains into carboxylate and hydroxyl groups, which makes it possible to follow the degradation process by monitoring the characteristics peaks by FTIR (stretching of carbonyl in the carboxylate group at 1600 cm^−1^ and bending of the O–H group at 975 cm^−1^) [33].

In Table 3, the most significant infrared peaks related to PLA are summarized from the literature. We use these peaks in order to characterize our electrospun mats during the degradation process.

**Figure 6:**
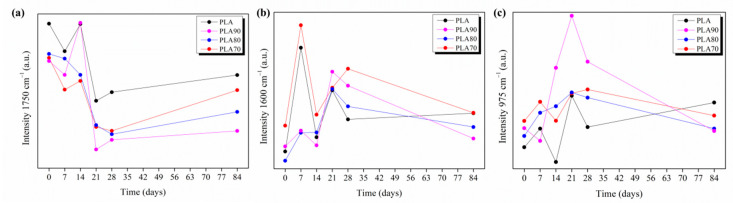
Evolution of intensity of 1750 cm^−1^ (**a**), 1600 cm^−1^ (**b**) and 975 cm^−1^ (**c**) peaks for neat PLA and each of the PLA:OLA electrospun samples by increasing the degradation days in PBS.

Thus, the FTIR spectra of each different PLA:OLA electrospun mat are studied at different immersion times in PBS media (0, 7, 14, 21, 28 and 84 days). In particular, the peaks’ evolutions at 1750 cm^−1^, 1600 cm^−1^ and 975 cm^−1^ for each immersion time are plotted in Figure 6 for PLA, PLA90, PLA80 and PLA70, respectively.

As we can see in Figure 6a, the variation of the intensity of the peak at 1750 cm^−1^, which is attributed to the amorphous carbonyl vibration, is studied. In all the samples, a slight drop at 7 days of degradation can be observed. As expected, following the hydrolytic reaction shown in Figure 5, this drop corresponds with an increase in the intensity of the 1600 cm^−1^ (Figure 6b) and 975 cm^−1^ peaks (Figure 6c). This fact indicates that the hydrolytic degradation started within the first week, which is is in accordance with the drastic reduction in the average diameter of nanofibers obtained by SEM.

At 14 days of immersion, the 1750 cm^−1^ peak rapidly grows up to the highest level for all the samples, which may be attributed to a significant conformational change. In fact, with the starting of the degradation process, molecules of water penetrated into the mats, inducing a less-ordered chain packaging, which can be attributed to the growth of carbonyl stretching the peak related to the amorphous phase.

From day 21 of degradation in PBS, the 1750 cm^−1^ peak drastically falls dawn. Meanwhile, the 1600 cm^−1^ and 975 cm^−1^ peaks increase up to the maximum level due to the existence of short degraded chains with carboxylate and hydroxyl end groups.

**Figure 7:**
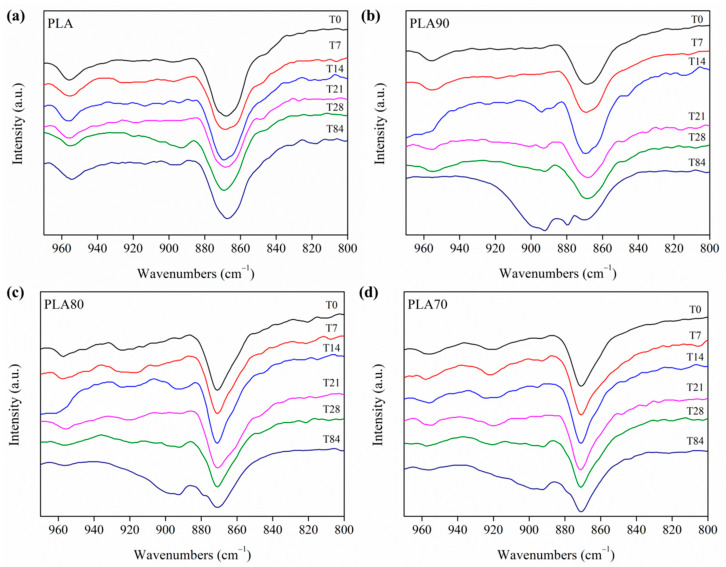
FTIR spectra in the 960–800 cm^−1^ range for (**a**) PLA and (**b**) PLA90, (**c**) PLA80 and (**d**) PLA70 samples after 0, 7, 14, 21, 28 and 84 days of immersion in PBS.

It is known that semicrystalline polymers, such as PLA, contain crystalline and amorphous regions. The long macromolecular chains’ segments are arranged more regularly and packed more strongly in the crystalline phase than in the amorphous one. Therefore, small molecules of water can attack the polymeric chains in the amorphous phase more easily and it is expected that the polymer chains in the amorphous regions will be fragmented earlier than those in the crystalline form. Previous studies have identified infrared absorption bands characteristic of the amorphous and crystalline phases in PLA [22], as also summarized in Table 3. Thus, the evolution of the infrared spectra can be applied to monitor the rearrangement of the crystalline phase in our systems, thus taking into account that absorption bands have fixed frequencies and others change between the amorphous and α crystalline phase. Therefore, in order to study the crystallinity evolution during the degradation process, three main peaks were followed by monitoring the FTIR spectra of each sample in the range of 960−800 cm^−1^ for 0, 7, 14, 21, 28 and 84 days of immersion in PBS (see Figure 7). These three bands were located at 860 and 955 cm^−1^ (both related to the amorphous phase of PLA) and 871 cm^−1^ (related to the crystalline α phase) (see Table 3). The variation in the crystallization is accompanied by a change in the shape of the absorption band at 871 cm^−1^. The 871 cm^−1^ band, attributed to the skeletal stretching and CH_3_ rocking of the crystalline α phase, can be observed in all the electrospun PLA-based systems as well as the 955 cm^−1^ band attributed to the amorphous phase; therefore, we can conclude that the crystallization of our samples is due to the presence of α crystals in all the mats. It is important to remark how the 955 cm^−1^ band, attributed to the amorphous phase, is retained constantly in the neat PLA sample during the entire degradation test, even at 84 days where the mat showed low crystallinity in comparison with the PLA:OLA electrospun samples. On the contrary, the remainder of the PLA:OLA electrospun samples showed a decrease in the 955 cm^−1^ band (amorphous phase) at 84 days. At this time, all the samples showed a high grade of α phase that can be associated with the change of intensity and shape of a broad peak at 871 cm^−1^. In accordance with the study of the evolution in carboxylate and hydroxyl group reported above, at time 14, a change of intensity and shape of the broad peak at 955 cm^−1^ was observed. As described previously, at this time of immersion, the degradation process provoked a conformational change in the crystals. Small molecules of water penetrated and plasticized the samples, which resulted in re-ordered chain packaging.

**Figure 8:**
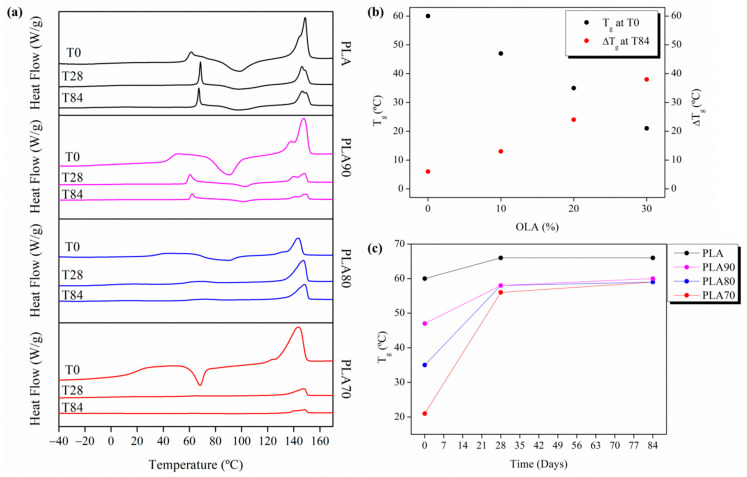
(**a**) DSC thermograms at T0, T28 and T84 of the different samples. (**b**) T_g_ at T0 for the different samples and their variation at T84. (**c**) The evolution of the T_g_ for neat PLA and each of the PLA:OLA electrospun samples after 0, 28 and 84 days of immersion in PBS.

The crystallization evolution studied by FTIR is corroborated by DSC results. In particular, the DSC thermograms for each of the PLA-based electrospun fiber mats at T0, T28 and T84 are plotted in Figure 8a. Figure 8b represents the evolution of their T_g_ during the degradation test, at T0 and its variation at T84, while the T_g_ evolution during the degradation process is reported in Figure 8c. Moreover, the X_c_ (%), calculated by DSC, is reported in Table 4. In particular, the degree of crystallinity increases by increasing the time of immersion, which shows the higher mobility of the polymer chains in the amorphous phase. Hydrolytic attack on the ester backbone decreases the PLA chain length by creating short polymeric chains. These short polymeric chains are more mobile and tend to crystallize, thus increasing the crystallinity of PLA. OLA is also an oligomer of PLA, that is, a PLA with a short chain. As can be seen, PLA70 shows the highest crystallinity after 84 days, probably due to the fact that the short chains produced from the hydrolytic degradation mixed with the high amount of plasticizer tend to recrystallize, increasing the crystalline phase. This fact can also be verified, thus taking into account the higher dimension of the crystals, reported in Table 4, at T84.

Furthermore, it can be seen that the addition of OLA decreases the T_g_ (Figure 8b) of the electrospun system, confirming the good compatibility between both polymers [20,27]. Moreover, due to the increasing of the degree of crystallinity, the reduced molecular mobility causes an increase in the T_g_, as can be seen in Figure 8c, from 60 °C to about 65 °C for neat PLA, from 47 °C for PLA90, from 35 °C for PLA80, and from 21 °C up to 55 °C for PLA70, in all the samples after 84 days of degradation in PBS. It is interesting to note that all the PLA:OLA electrospun mats seem to reach a plateau at about 55 °C, independently of the amount of OLA added into the system.

Since the degree of crystallinity of neat PLA seems to not change significantly, it is clear that the addition of OLA affects the crystallization and the degradation process of the polymeric chains.

**Figure 9:**
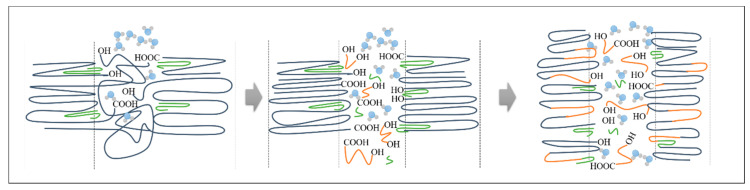
Schematic representation of the hydrolysis process in the amorphous and crystalline regions. Long PLA chains are represented with blue lines, short PLA chains are in orange, and OLA is represented in green.

It has been reported that the hydrolysability is faster for the polymers with a high degree of crystallinity than for the amorphous ones [38] due to the larger density of hydrophilic terminal groups (carboxyl –COOH and hydroxyl –OH) in the amorphous region in between the crystalline zones [39,40]. Additionally, the high number of terminal groups causes the recrystallization process to occur, which increases the diffusion of water as well [41]. This enhanced water diffusion accelerates the hydrolysis of the amorphous region in the crystalline polymer in comparison with the amorphous one [39], leading to a faster degradation, as can be observed in Figure 3, where PLA70, the sample with the highest degree of crystallinity, is the first one to achieve total disintegration. In order to better understand this phenomenon, the hydrolysis process between the amorphous and the crystalline regions of our systems is represented in Figure 9. To facilitate the visualization of the polymeric chains during the different degradation stages, different colors are used; in particular, long PLA chains are represented with blue lines, short PLA chains are in orange, and OLA is represented in green.

Therefore, for all the samples, the degradation process can be divided into three steps. In the first step, during the first week of degradation in PBS, the water molecules broke the ester bond of the long polymeric chains in the amorphous phase (Stage I). Then, during the second week, a reorganization of the short chain occurred, attributed to an increase in amorphous carboxyl peak (Stage II). Finally, after day 14, the polymeric chains present in the amorphous phases showed a drastically hydrolytic degradation due to the diffusion of water molecules within the degraded matrix, which carried with it an increase in crystallinity for all the samples (Stage III).

**Figure 10:**
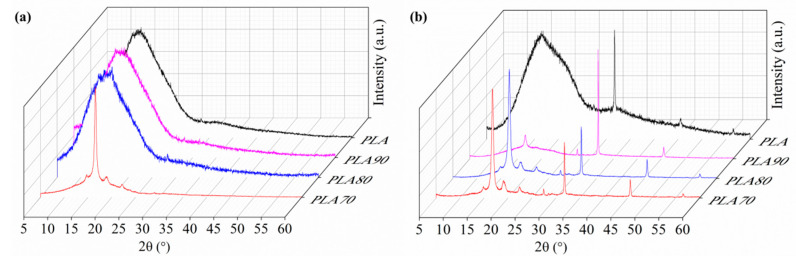
XRD pattern of neat PLA and each of the PLA:OLA electrospun samples: (**a**) before starting the degradation test and (**b**) after 84 days of immersion in PBS.

XRD provides an ideal method to monitor changes in the crystallization evolution of polymer during the degradation process. The XRD patterns of the PLA and PLA:OLA electrospun fiber mats are reported in Figure 10.

As can be seen, before starting the degradation test, at T0, neat PLA demonstrates an amorphous structure; meanwhile, by increasing the amount of OLA, the degree of crystallinity increases. For PLA70, strong peaks at 2θ = 16.5, 19.1 and 22.5° clearly appeared, which were attributed to the [200/110], [203] and [015] crystallographic planes of α crystals in PLA [10,11]. As can be seen, after 84 days of immersion in PBS, these peaks appear for the rest of the PLA:OLA electrospun samples. In Table 4, the characteristic peaks obtained for the PLA-based samples at the different degradation times are reported. Moreover, the crystal dimension (D), calculated by the Scherrer Equation, is reported in order to confirm the crystallization process evolution. At T0, only PLA70 shows crystalline peaks in different crystallographic plane directions, with a total degree of crystallinity of 45%. At T84, PLA70 shows the highest crystallinity, while PLA90 and PLA80 show new crystalline peaks, thus increasing their crystallinity. For pristine PLA, no crystallographic planes of α crystals can be appreciated after 84 days of immersion.

Moreover, the degree of crystallinity obtained by DSC is in good agreement with the same calculated by XRD, reported in the Table 4. It is quite interesting that neat PLA from DSC at T84 shows a small degree of crystallinity that is not obtained by XRD. This fact can be attributed to the mineralization process and to the presence of the crystalline structure of NaCl in the degraded electrospun mats.

The mechanism associated with the hydrolysis of the ester group in a neutral or acid medium is different from that in an alkaline medium [16]. This hydrolytic degradation of PLA has been reported to occur by two different mechanisms, which have a crucial impact on the performance of PLA-based materials as implants in the human body [16]. For this reason, it is important to study the pH evolution during the degradation test.

**Figure 11:**
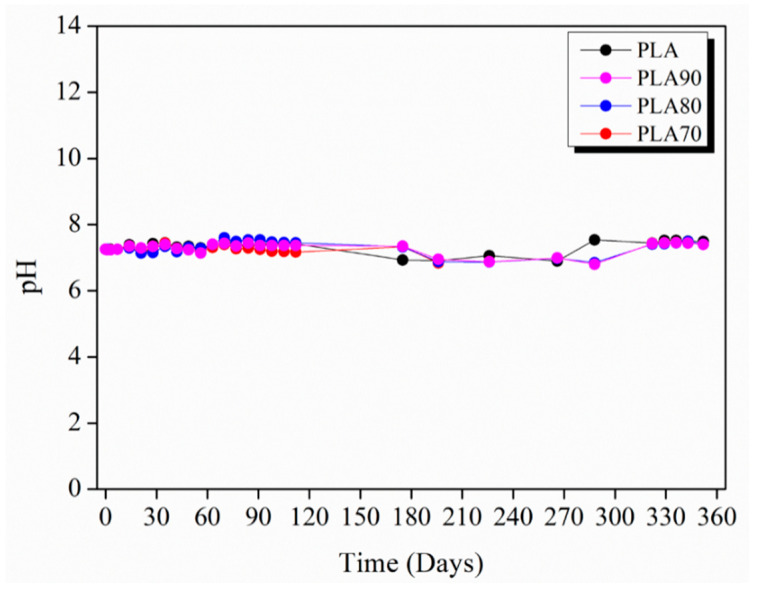
pH values measured weekly for neat PLA and each of the PLA:OLA electrospun samples up to 352 days of immersion in PBS.

Figure 11 shows the pH values measured every 7 days for neat PLA and PLA90, PLA80 and PLA70 samples up to 352 days of immersion in PBS. As can be appreciated, the measurements in PBS reveal that a neutral pH (from 7.0 to 7.5) is retained during the whole degradation test of almost 1 year of duration. The behavior of pH evolution in all the samples is quite similar and, during all the degradation tests, all of the samples present a neutral pH value.

**Figure 12:**
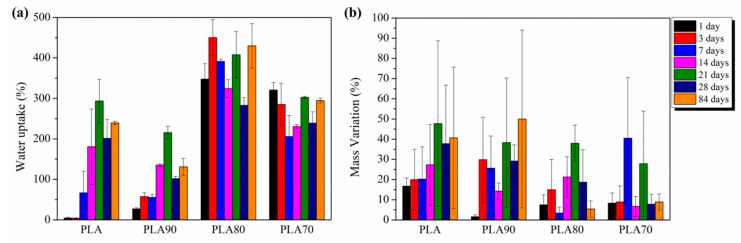
(**a**) Water uptake (%) and (**b**) mass variation (%) for neat PLA and each of the PLA:OLA electrospun samples after different amounts of days of immersion in PBS.

Figure 12 shows the degradation evolution of electrospun PLA-based samples until 84 days of immersion in PBS in terms of water uptake and mass variation. During the first days of degradation (1 and 3), electrospun neat PLA retains less water than plasticized systems. By increasing the amount of OLA, the water retention increased during the first days. At 7 days of immersion, water uptake increased up to 70% and 56% for PLA and PLA90, respectively. For PLA70 and PLA80, this rise is higher, reaching values of 206% and 392%, respectively. The degradation test was carried out at 37 °C, which is slightly higher for T_g_ values of PLA70 and PLA80 samples (see Figure 8). The polymeric chains present more mobility at this temperature; therefore, the water molecules from PBS can quickly get into the polymeric structure and a higher amount of water can be accumulated in the sample structure.

From the mass variation point of view, it is important to remark on how the piece of mat for each formulation was gaining mass as the degradation time increased due to mineralization process and deposition of NaCl crystals, as mentioned before. The high porosity values of electrospun mats allowed the PBS media to flow through the electrospun fibers, resulting in a transfer of ions from the solution to the surface of the PLA-based electrospun mats, which can hardly be eliminated in order to measure the decrease in mass in degraded PLA-based mats. Due to the significant measurement errors, we can conclude, in our case, that the study of mass variation is not adequate to characterize the degradation process of electrospun mats formed by fibers and holes between the fibers’ networks.

## 4. Conclusions

Electrospun fiber mats based on PLA are successfully obtained and characterized. In particular PLA is plasticized with OLA in different amounts, changing the final properties in terms of morphology, T_g_ and crystallinity. We focused on the hydrolytic degradation process and we can conclude that, on the one hand, the addition of OLA increases the hydrolytic degradation rate of PLA electrospun fiber mats and, on the other hand, that by adding different amounts of OLA, the time of degradation in PBS of the electrospun fibers mat can be modulated over the course of a year. This fact is very interesting considering the potential application of such materials as biomedical implants. In particular, when the amount of OLA is increased, the diameter of the electrospun fibers decreases during the degradation process. The degree of crystallinity of the electrospun fiber mats is highly affected not only by the presence but also by the amount of OLA at T0 as well as during the entirety of the degradation process. In fact, by degrading PLA-based electrospun materials in PBS, crystallinity increases, and the dimension of the α crystals increases due to the selective hydrolytic attack of amorphous phase and the consequent recrystallization of their short chains. Moreover, as expected, the pH is constantly maintained at a neutral range for almost a year, which is the duration of our degradation process.

## Figures and Tables

**Table 1:** PLA:OLA electrospun formulations and average diameter value of fibers at T0.

Sample	PLA (%)	OLA (%)	Diameter (nm) at T0
PLA	100	0	904 ± 33
PLA90	90	10	875 ± 63
PLA80	80	20	689 ± 34
PLA70	70	30	486 ± 59

**Table 2:** Element composition obtained by EDX analysis for neat PLA and each PLA:OLA samples after 0, 28 and 84 days of immersion in PBS: T0, T28 and T84, respectively.

		T0	T28	T84
	Element	At (%)	At (%)	At (%)
PLA	C	58.98	61.33	60.79
	O	40.97	33.67	36.05
	Na	0.00	3.09	1.86
	Cl	0.00	1.76	1.21
PLA90	C	58.02	60.62	59.88
	O	41.98	35.42	38.13
	Na	0.00	2.18	1.22
	Cl	0.00	1.44	0.72
PLA80	C	59.77	61.74	60.95
	O	40.23	33.74	36.05
	Na	0.00	2.28	1.54
	Cl	0.00	2.07	1.34
PLA70	C	58.05	62.47	62.57
	O	41.95	33.92	30.52
	Na	0.00	1.76	3.38
	Cl	0.00	1.76	3.26

**Table 3:** The most significant infrared bands related to different phases and assignment of PLA.

IR Frequency (cm^−1^)	Phase Form	Assignment	Ref. No.
1750		Stretching of amorphous carbonyl (–C=O) group.	[22,33]
1600		Stretching of carbonyl (–C=O) in carboxylate group.	[22,33]
975		Bending of O–H group.	[22,33]
955	Amorphous		[35,36,37]
908	β		[22,36]
871	α		[22,37]
860	Amorphous		[22,35]

**Table 4:** Results of XRD analysis for neat PLA and each of the PLA:OLA electrospun samples for T0 and T84.

	Sample	*hkl*	2θ (°)	D (nm)	*X*_c_ (%) XRD	*X*_c_ (%) DSC
T0	PLA	-	-	-	-	0.8
	PLA90	-	-	-	-	1
	PLA80	-	-	-	-	1
	PLA70	010	15.1	3.2	45.1	27.4
		200/110	16.6	19.8		
		203	18.7	3.9		
		015	22.1	17.7		
		207	27.5	64.4		
		216	29.0	20.1		
T84	PLA	-	-	-	-	7
	PLA90	010	-	-	10.4	10
		200/110	16.6	18.4		
		203	-	-		
		015	-	-		
		207	-	-		
		216	27.5	77.5		
	PLA80	010	15.1	4.4	47.5	38
		200/110	16.8	17.5		
		203	19.1	9.8		
		015	22.4	19.1		
		207	27.5	55.7		
		216	29.2	10.5		
	PLA70	010	15.2	4.0	52.1	47
		200/110	16.8	18.9		
		203	19.1	12.2		
		015	22.4	21.1		
		207	27.5	64.4		
		216	29.2	23.4		

## References

[1] Leonés A., Lieblich M., Benavente R., Gonzalez J.L., Peponi L. (2020). Potential applications of magnesium-based polymeric nanocomposites obtained by electrospinning technique. Nanomaterials.

[2] Arrieta M.P., López J., López D., Kenny J.M., Peponi L. (2015). Development of flexible materials based on plasticized electrospun PLA-PHB blends: Structural, thermal, mechanical and disintegration properties. Eur. Polym. J..

[3] Leonés A., Mujica-Garcia A., Arrieta M.P., Salaris V., Lopez D., Kenny J.M., Peponi L. (2020). Organic and Inorganic PCL-Based Electrospun Fibers. Polymers.

[4] Jiang S., Chen Y., Duan G., Mei C., Greiner A., Agarwal S. (2018). Electrospun nanofiber reinforced composites: A review. Polym. Chem..

[5] Peponi L., Mújica-García A., Kenny J.M. (2015). Electrospinning of PLA. Hyperbranched Polym. Macromol. Determ. Linear Chain. Dendrimer Struct..

[6] Arrieta M., Díez García A., López D., Fiori S., Peponi L. (2019). Antioxidant Bilayers Based on PHBV and Plasticized Electrospun PLA-PHB Fibers Encapsulating Catechin. Nanomaterials.

[7] Torres-Giner S., Echegoyen Y., Teruel-Juanes R., Badia J.D., Ribes-Greus A., Lagaron J.M. (2018). Electrospun Poly(ethylene-co-vinyl alcohol)/Graphene Nanoplatelets Composites of Interest in Intelligent Food Packaging Applications. Nanomaterials.

[8] Huang Z.-M., Zhang Y.-Z., Kotaki M., Ramakrishna S. (2003). A review on polymer nanofibers by electrospinning and their applications in nanocomposites. Compos. Sci. Technol..

[9] Sonseca A., Peponi L., Sahuquillo O., Kenny J.M., Giménez E. (2012). Electrospinning of biodegradable polylactide/hydroxyapatite nanofibers: Study on the morphology, crystallinity structure and thermal stability. Polym. Degrad. Stab..

[10] Varesano A., Rombaldoni F., Mazzuchetti G., Tonin C., Comotto R. (2010). Multi-jet nozzle electrospinning on textile substrates: Observations on process and nanofibre mat deposition. Polym. Int..

[11] Sun G., Sun L., Xie H., Liu J. (2016). Electrospinning of nanofibers for energy applications. Nanomaterials.

[12] Lv D., Wang R., Tang G., Mou Z., Lei J., Han J., De Smedt S., Xiong R., Huang C. (2019). Ecofriendly Electrospun Membranes Loaded with Visible-Light-Responding Nanoparticles for Multifunctional Usages: Highly Efficient Air Filtration, Dye Scavenging, and Bactericidal Activity. ACS Appl. Mater. Interfaces.

[13] Jiang S., Uch B., Agarwal S., Greiner A. (2017). Ultralight, Thermally Insulating, Compressible Polyimide Fiber Assembled Sponges. ACS Appl. Mater. Interfaces.

[14] Arrieta M.P., Gil A.L., Yusef M., Kenny J.M., Peponi L. (2020). Electrospinning of PCL-based blends: Processing optimization for their scalable production. Materials.

[15] Rowe M.J., Kamocki K., Pankajakshan D., Li D., Bruzzaniti A., Thomas V., Blanchard S.B., Bottino M.C. (2016). Dimensionally stable and bioactive membrane for guided bone regeneration: An in vitro study. J. Biomed. Mater. Res. Part. B Appl. Biomater..

[16] Araque-Monrós M.C., Vidaurre A., Gil-Santos L., Gironés Bernabé S., Monleón-Pradas M., Más-Estellés J. (2013). Study of the degradation of a new PLA braided biomaterial in buffer phosphate saline, basic and acid media, intended for the regeneration of tendons and ligaments. Polym. Degrad. Stab..

[17] Arrieta M.P., Peponi L. (2017). Polyurethane based on PLA and PCL incorporated with catechin: Structural, thermal and mechanical characterization. Eur. Polym. J..

[18] Arrieta M.P., López J., López D., Kenny J.M., Peponi L. (2016). Biodegradable electrospun bionanocomposite fibers based on plasticized PLA–PHB blends reinforced with cellulose nanocrystals. Ind. Crops Prod..

[19] Navarro-Baena I., Marcos-Fernandez A., Kenny J.M., Peponi L. (2014). Crystallization behavior of diblock copolymers based on PCL and PLLA biopolymers. J. Appl. Crystallogr..

[20] Burgos N., Tolaguera D., Fiori S., Jiménez A. (2014). Synthesis and Characterization of Lactic Acid Oligomers: Evaluation of Performance as Poly(Lactic Acid) Plasticizers. J. Polym. Environ..

[21] da Silva D., Kaduri M., Poley M., Adir O., Krinsky N., Shainsky-Roitman J., Schroeder A. (2018). Biocompatibility, biodegradation and excretion of polylactic acid (PLA) in medical implants and theranostic systems. Chem. Eng. J..

[22] Ribeiro C., Sencadas V., Costa C.M., Gómez Ribelles J.L., Lanceros-Méndez S. (2011). Tailoring the morphology and crystallinity of poly(L-lactide acid) electrospun membranes. Sci. Technol. Adv. Mater..

[23] Baraúna G., Coraça-Huber D.C., Duek E.A. (2012). de R. In vitro degradation of Poly-L-co-D, L-lactic acid membranes. Mater. Res..

[24] Nim B., Sreearunothai P., Petchsuk A., Opaprakasit P. (2018). Preparation of TiO2-loaded electrospun fibers of polylactide/poly(vinylpyrrolidone) blends for use as catalysts in epoxidation of unsaturated oils. J. Nanoparticle Res..

[25] Mujica-Garcia A., Navarro-Baena I., Kenny J.M., Peponi L. (2014). Influence of the Processing Parameters on the Electrospinning of Biopolymeric Fibers. J. Renew. Mater..

[26] Carrasco-Hernandez S., Gutierrez J., Peponi L., Tercjak A. (2017). Optimization of the electrospinning processing-window to fabricate nanostructured PE-b-PEO and hybrid PE-b-PEO/EBBA fibers. Polym. Eng. Sci..

[27] Leonés A., Sonseca A., López D., Fiori S., Peponi L. (2019). Shape memory effect on electrospun PLA-based fibers tailoring their thermal response. Eur. Polym. J..

[28] Peponi L., Navarro-Baena I., Báez J.E., Kenny J.M., Marcos-Fernández A. (2012). Effect of the molecular weight on the crystallinity of PCL-b-PLLA di-block copolymers. Polymer.

[29] Navarro-Baena I., Kenny J.M., Peponi L. (2014). Crystallization and thermal characterization of biodegradable tri-block copolymers and poly(ester-urethane)s based on PCL and PLLA. Polym. Degrad. Stab..

[30] Arrieta M.P., Perdiguero M., Fiori S., Kenny J.M., Peponi L. (2020). Biodegradable electrospun PLA-PHB fibers plasticized with oligomeric lactic acid. Polym. Degrad. Stab..

[31] Rodriguez-Navarro C., Linares-Fernandez L., Doehne E., Sebastian E. (2002). Effects of ferrocyanide ions on NaCl crystallization in porous stone. J. Cryst. Growth.

[32] Martin O., Avérous L. (2001). Poly(lactic acid): Plasticization and properties of biodegradable multiphase systems. Polymer.

[33] Arrieta M.P., López J., López D., Kenny J.M., Peponi L. (2016). Effect of chitosan and catechin addition on the structural, thermal, mechanical and disintegration properties of plasticized electrospun PLA-PHB biocomposites. Polym. Degrad. Stab..

[34] Dias J.C., Ribeiro C., Sencadas V., Botelho G., Ribelles J.L.G., Lanceros-Mendez S. (2012). Influence of fiber diameter and crystallinity on the stability of electrospun poly(l-lactic acid) membranes to hydrolytic degradation. Polym. Test..

[35] Zhang J., Tsuji H., Noda I., Ozaki Y. (2004). Weak Intermolecular Interactions during the Melt Crystallization of Poly(L -lactide) Investigated by Two-Dimensional Infrared Correlation Spectroscopy. J. Phys. Chem. B.

[36] Zhang J., Duan Y., Sato H., Tsuji H., Noda I., Yan S., Ozaki Y. (2005). Crystal Modifications and Thermal Behavior of Poly(L -lactic acid) Revealed by Infrared Spectroscopy. Macromolecules.

[37] Vasanthan N., Ly O. (2009). Effect of microstructure on hydrolytic degradation studies of poly (l-lactic acid) by FTIR spectroscopy and differential scanning calorimetry. Polym. Degrad. Stab..

[38] Tsuji H., Del Carpio C.A. (2003). In Vitro Hydrolysis of Blends from Enantiomeric Poly(lactide)s. 3. Homocrystallized and Amorphous Blend Films. Biomacromolecules.

[39] Tsuji H., Mizuno A., Ikada Y. (2000). Properties and morphology of poly(L-lactide). III. Effects of initial crystallinity on long-termin vitro hydrolysis of high molecular weight poly(L-lactide) film in phosphate-buffered solution. J. Appl. Polym. Sci..

[40] Gorrasi G., Pantani R., Di Lorenzo M., Androsch R. (2017). Hydrolysis and Biodegradation of Poly(lactic acid). Synthesis, Structure and Properties of Poly(Lactic Acid).

[41] Cifuentes S.C., Lieblich M., Saldaña L., González-Carrasco J.L., Benavente R. (2019). In vitro degradation of biodegradable polylactic acid/Mg composites: Influence of nature and crystalline degree of the polymeric matrix. Materialia.

